# Nasal delivery of Fasudil‐modified immune cells exhibits therapeutic potential in experimental autoimmune encephalomyelitis

**DOI:** 10.1111/cns.13111

**Published:** 2019-02-18

**Authors:** Shang‐De Guo, Chun‐Yun Liu, Jing‐Wen Yu, Zhi Chai, Qing Wang, Xi‐Ting Mi, Guo‐Bin Song, Yan‐Hua Li, Peng‐Wei Yang, Ling Feng, Bao‐Guo Xiao, Cun‐Gen Ma

**Affiliations:** ^1^ Department of Neurology, Institute of Brain Science, Medical School Shanxi Datong University Datong China; ^2^ Shanxi Key Laboratory of Inflammatory Neurodegenerative Diseases, Institute of Brain Science Shanxi Datong University Datong China; ^3^ Research Center of Neurobiology Shanxi University of Traditional Chinese Medicine Taiyuan China; ^4^ Institute of Neurology Huashan Hospital Institutes of Brain Science and State Key Laboratory of Medical Neurobiology Fudan University Shanghai China

**Keywords:** cell therapy, experimental autoimmune encephalomyelitis, Fasudil‐modified mononuclear cells, nasal delivery

## Abstract

**Aim:**

Multiple sclerosis (MS) is a relapsing‐remitting inflammatory demyelinating disease that requires long‐term treatment. Although Rho kinase inhibitor Fasudil shows good therapeutic effect in experimental autoimmune encephalomyelitis (EAE), an animal model of MS, certain side effects may limit its clinical use. This study aimed at observing the therapeutic potential of Fasudil‐modified encephalitogenic mononuclear cells (MNCs) via nasal delivery in EAE and exploring possible mechanisms of action.

**Methods:**

Experimental autoimmune encephalomyelitis was induced with myelin oligodendrocyte glycoprotein 35‐55 in C57BL/6 mice, and encephalitogenic MNCs were treated with Fasudil in vitro. Mice received 3 × 10^6^ cells/10 μL per nasal cavity on day 3 and 11 postimmunization, respectively.

**Results:**

Fasudil‐modified MNCs reduced clinical severity of EAE, improved demyelination, and decreased inflammatory cells in spinal cords. Immunohistochemical results indicated that CD4^+^ T cells and CD68^+^ macrophages were barely detected in Fasudil‐MNCs group. Fasudil‐modified MNCs decreased CD4^+^IFN‐γ^+^ and CD4^+^IL‐17^+^ T cells, increased CD4^+^IL‐10^+^ T cells, restrained M1 markers CD16/32, CCR7, IL‐12, CD8a, enhanced M2 markers CD206, CD200, CD14 in spleen. Fasudil‐modified MNCs inhibited the activation of inflammatory signaling p‐NF‐kB/P38, accompanied by the decrease of COX‐2 and the increase of Arg‐1 in spinal cord, as well as the reduction of IL‐17, TNF‐α, IL‐6 and the elevation of IL‐10 in cultured supernatant of splenocytes. Fasudil‐modified MNCs enhanced the levels of neurotrophic factors BDNF and NT‐3 in spinal cord.

**Conclusion:**

Our results indicate that intranasal delivery of Fasudil‐modified MNCs have therapeutic potential in EAE, providing a safe and effective cell therapeutic strategy to MS and/or other related disorders.

## 1 INTRODUCTION

Multiple sclerosis (MS) is a chronic inflammatory demyelinating disease of the central nervous system (CNS), which leads to neurological impairment and most often affects young adults, with heavy burdens for the family and society. So far, no clinically effective therapy has been established to prevent neuronal damage in MS. The drugs approved by Food and Drug Administration (FDA) for the treatment of MS, such as interferon‐β, glatiramer acetate and Fingolimod, can alleviate the symptoms to a certain extent and reduce relapse frequency, whereas they are not effective for all patients of MS and do not influence the irreversible dysfunction and disease progression.^1^, ^2^ It is particularly urgent to find and study effective treatment strategies for MS patients with small side effect and low cost.

Experimental autoimmune encephalomyelitis (EAE), an ideal mouse model of MS, has many similarities in clinical manifestations, immunological and pathological processes with MS patients. The immunopathogenesis of MS/EAE involves the activation of peripheral immune system, the migration of activated immune cells to CNS, the formation of central immunological microenvironment and the injury of myelin and/or nerve tissue by hazardous substance.^3^, ^4^ However, the activation of peripheral immune cells is the initiating factor in the development of MS/EAE, which can lead to subsequent immune attacks and tissue damage.^5^ A lot of studies have found that interferon‐γ (IFN‐γ) producing T helper 1 (Th1) cells and interleukin‐17 (IL‐17) producing Th17 cells are the main participators in the development of EAE, which is closely related to neuroinflammation and myelin loss.^6^, ^7^ Th2 T cells act as immunomodulatory cells and possess the effect of antiinflammatory and myelin protection, which can improve the severity of EAE.^8^ Macrophages, an important antigen presenting cells of immune system, participate in the induction and regulation of inflammatory microenvironment. Classically activated macrophages (M1) can activate Th1 and Th17 of T cells and enlarge immunological response. Alternative activated macrophages (M2) can take part in mediating the differentiation of Th2 cells and CD4^+^CD25^+^ regulatory T cells, which contributes to the myelin regeneration and neuronal survival.^9^, ^10^, ^11^

Rho‐kinase (ROCK), is expressed both centrally and peripherally where it is involved in fundamental cellular processes, including differentiation, migration, proliferation and survival.^12^, ^13^ A series of studies have discovered that many neurodegenerative diseases, such as MS, Alzheimer's disease and Parkinson's disease, have the activation of ROCK signaling pathway that affects the occurrence and development of these diseases.^14^, ^15^, ^16^ Therefore, the inhibition of ROCK activity is considered as a potential drug target for the treatment of neurodegenerative diseases. Fasudil (1‐[5‐isoquinolinesulphonyl]‐homopiperazine), a selective ROCK inhibitor, has been widely used clinically since 1995 for the treatment of subarachnoid hemorrhage in Japan. Previous investigations from our group and other labs have indicated that Fasudil ameliorates the clinical severity of EAE in different models, accompanied by reduced demyelination and inhibition of neuroinflammation.^17^, ^18^

Although Fasudil shows good therapeutic effects in EAE, the following factors restrict its clinical application in MS patients: (a) rapid and obvious vasodilatation; (b) poor oral bioavailability and no oral drug; (c) smaller safety window for long‐term use.^19^ In recent years, cellular immunotherapy has become a hot spot in the field of neurological disorders and has made great progress, which has the advantages including autologous transplantation, economic security, no ethical problems and tumorigenicity.^20^, ^21^ Intranasal administration is a safe and effective delivery in the treatment of CNS diseases.^22^ Previous studies show that Fasudil also influence the polarization of T cells and macrophages.^23^ In this study, we try to explore the therapeutic effect of encephalitogenic mononuclear cells (MNCs) treated with Fasudil in vitro and possible mechanisms of action in the treatment of EAE by intranasal delivery. MNCs treated with Fasudil in vitro, via direct intranasal delivery, may represent a novel, simple, noninvasive strategy for the treatment of MS.

## 2 MATERIALS AND METHODS

### 2.1 Animals

Female C57BL/6 mice (8‐10 weeks and 18‐20 g) were obtained from Vital River Laboratory Animal Technology Co. Ltd. (Beijing, China) and maintained under pathogen‐free conditions in a reversed light/dark cycle (25 ± 2°C). All experiments were conducted in accordance with the International Council for Laboratory Animal Science guidelines. The study was approved by the Ethics Committee of Shanxi Datong University, Datong, China.

### 2.2 Preparation of encephalitogenic MNCs in vivo

Chronic EAE was induced by subcutaneous immunization on the upper dorsal flanks with 300 μg of myelin oligodendroglia glycoprotein 35‐55 (MOG_35‐55_) (CL Bio‐Scientific. Company, Xi'an, China) in Freund's complete adjuvant (Sigma, St Louis, MO) supplemented with 3 mg/mL of Mycobacterium tuberculosis H37Ra (BD Difco, Franklin Lakes, NJ) (300 μg/mice). Mice were intraperitoneally injected with 400 ng of pertussis toxin (Enzo Life Sciences, Farmingdale, NY) at the same time as immunization and again 48 hour later. On day 9 postimmunization (p.i.), mice were sacrificed and spleens were removed under aseptic conditions. Suspension of MNCs from spleen was prepared by grinding the spleen through a 40‐μm nylon cell strainer (BD, Franklin Lakes, NJ) in medium. Erythrocytes in the suspension were osmotically lysed. Cells were then washed three times and re‐suspended in medium. Cells were adjusted to 3×10^6^/mL. Subsequently, encephalitogenic MNCs were treated with Fasudil (15 μg/mL) or phosphate buffered solution (PBS) in the presence of IL‐2 (20 ng/mL) and MOG_35‐55_ (10 μg/mL) for 72 hour at 37°C with 5% CO_2_.

### 2.3 Induction of EAE and intranasal administration of MNCs

Mice were divided into two groups: Fasudil‐MNCs (n = 10) and PBS‐MNCs (n = 10). After washing, MNCs from above preparation were resuspended at 6 × 10^6^ cells in normal saline (NS) and administrated by bilateral intranasal instillation into mice immunized with MOG_35‐55。_For intranasal administration, mice were lightly anesthetized with diethylether, and then received 3 × 10^6^ cells/10 μl per nasal cavity on day 3 p.i. and day 11 p.i., respectively. Following cell transplantation, clinical score and body weight were evaluated every other day in a blinded fashion by at least two investigators. Clinical score of EAE was graded according to the following criteria: 0. healthy; 1. limp tail; 2. ataxia and/or paresis of hindlimbs; 3. paralysis of hindlimbs and/or paresis of forelimbs; 4. tetraparalysis; 5. moribund or death. All animal experiments were repeated three times.

### 2.4 Histology and immunohistochemistry

On day 28 p.i., mice were perfused with NS and 4% buffered paraformaldehyde. Spinal cord was sliced (10 μm) and pathological change was detected by hematoxylin & eosin staining (H&E), luxol fast blue staining, and immunohistochmistry. The inflammatory invasion in spinal cord was evaluated by Image‐Pro Plus software according to the previous description^24^: 0. no inflammation cell infiltration; 1. inflammatory cell infiltration is limited only around the perivascular and spinal meninges; 2. mild inflammatory cell infiltration in the parenchyma of the spinal cord (1‐10/field); 3. moderate inflammatory cell infiltration in the parenchyma of the spinal cord (10‐100/field); 4. heavy inflammatory cell infiltration in the parenchyma of the spinal cord (>100/field). Total white matter in luxol fast blue was manually outlined, and pixel area (%) of demyelination in total white matter was calculated by Image‐Pro Plus software.

For immunohistochemistry, sections were blocked with 1% albumin from bovine serum (BSA) (Serotec, Bicester, UK) for 30 minutes and incubated with anti‐CD4 (1:1000; clone A60; Sigma), anti‐CD68 (1:1000; Serotec) and anti‐microtubule associated protein 2 (MAP2) (1:200; Millipore, Bedford, MA, USA) at 4°C overnight, followed by the corresponding secondary antibodies at room temperature (RT) for 2 hour. As a negative control, additional sections were treated similarly, but the primary antibodies were omitted. The expressions of CD4^+^ T cells, CD68^+^ macrophages and MAP2 were determined by Image‐Pro Plus software.

### 2.5 Flow cytometry analysis

On day 28 p.i., mice were sacrificed and spleens were removed under aseptic conditions. MNCs were prepared as above described stained for 20 minutes at RT in 1% BSA‐PBS buffer with the following panel of antibodies: FITC‐CD4 (eBioscience, San Diego, CA, USA) and PE‐CD25 (eBioscience), Alexa Fluor 488‐anti‐CD11b (eBioscience) and PE‐CD16/32, PE‐CD206, PE‐CD11c, PE‐CD40, PE‐CD8a, PE‐CD14, PE‐CD200 (eBioscience). For intracellular staining, MNCs were stained for 20 minutes at RT in 0.3% saponin/1% BSA‐PBS buffer with the following panel of antibodies: FITC‐CD4 and PE‐IL‐10, PE‐transforming growth factor‐β (TGF‐β), PE‐IFN‐γ, PE‐IL‐17 (eBioscience), Alexa Fluor 488‐anti‐CD11b and PE‐IL‐12, PE‐chemokine receptor7 (CCR7) (eBioscience). At least 10 000 events were collected using flow cytometer (BD Biosciences, Franklin Lakes, NJ, USA) and data were analysed using CellQuest software (BD Biosciences).

### 2.6 Western blot analysis

On day 28 p.i., mice were perfused with NS and spinal cords were homogenized with a microcontent motoroperated tissue homogenizer (Kimble Kontes, Vineland, NJ, USA), using protein extraction kit (Millipore) supplemented with a cocktail of protease inhibitors. The homogenates were centrifuged at 20 000 *g* for 20 minutes at 4°C, and the supernatants were collected. Protein extract (20 μg) were separated by SDS‐PAGE and electroblotted onto nitrocellulose membrane (Immobilon‐P; Millipore). After blocking with 5% nonfat dry milk, the membranes were incubated at 4°C overnight with the following antibodies: antiinducible nitric oxide synthase (iNOS) (1:200; Cayman Chemicals Company, Ann Arbor, MI, USA), anti‐arginase‐1 (Arg‐1) (1:300; Cayman Chemicals Company), anti‐cyclooxygenase‐2 (COX‐2) (1:1000; Abcam, Cambridge, UK), antitoll like receptor 2 (TLR2) (1:1000; Danvers, MA, USA), anti‐p‐nuclear factor kappa B (p‐NF‐κB) (1:1000; Epitomics, Burlingame, CA, USA), anti‐P38 (1:1000; Abcam), antibrain derived neurotrophic factor (BDNF) (1:1000; Promega, Madison, WI), anti‐neurotrophin‐3 (NT‐3) (1:1000; Epitomics) and anti‐glyceraldehyde‐3‐phosphate dehydrogenase (GAPDH) (1:1000; Epitomics) overnight at 4°C. Bands were visualized by horseradish peroxidase‐conjugated secondary antibodies and chemiluminescence (ECL) kit under ECL system (GE Healthcare Life Sciences, Niskayuna, NY, USA).

### 2.7 Cytokines by enzyme linked immunosorbent assay

On day 28 p.i., mice were sacrificed and spleens were removed under aseptic conditions. Splenic MNCs (6 × 10^5^/mL) were cultured for 48 hour at 37°C in the presence of MOG_35‐55_ (10 μg/mL). Supernatants were collected and measured for cytokine concentrations of IL‐17, IL‐10 (eBioscience Inc), IL‐6, tumor necrosis factor (TNF‐α) (PeproTech Inc., Hawthorne, NJ, USA) and IL‐1β (Invitrogen Inc., Carlsbad, CA, USA) using sandwich enzyme linked immunosorbent assay (ELISA) kits in accordance with the manufacturer's instructions. The quantitation of cytokines was calculated by reference to standard curves. Determinations were performed in triplicate and results were expressed as pg/mL.

### 2.8 Statistical analysis

GraphPad Prism software (Cabit Information Technology Co., Ltd., Shanghai, China) was used for statistical analysis. The data of clinical mean score was analysed with the Mann‐Whitney *U* test; other data were analysed with Student's *t* test. A statistically significant difference was assumed at *P* < 0.05.

## 3 RESULTS

### 3.1 Fasudil‐modified MNCs ameliorates severity of EAE via nasal delivery

**Figure 1:**
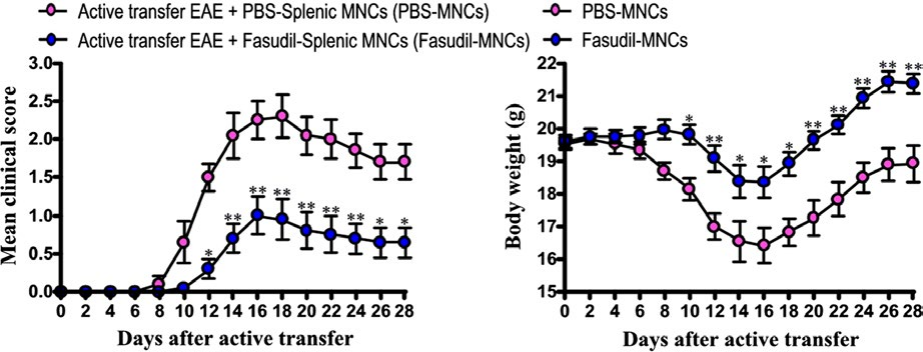
Fasudil‐treated mononuclear cells (MNCs) ameliorate the severity of experimental autoimmune encephalomyelitis (EAE). Chronic EAE was induced in C57BL/6 mice with MOG_35‐55_. Fasudil‐treated MNCs were administrated by bilateral intranasal instillation with 3 × 10^6^ cells/unilateral nasal cavity on day 3 postimmunization (p.i.) and 11 p.i. Phosphate buffered solution (PBS)‐treated MNCs were set up as control in a similar manner (n = 10 each group). Mean clinical score and body weight were recorded. Differences are analyzed by Mann‐Whitney *U* test after nonparametric Kruskal‐Wallis test or Student's *t*‐test. *P < 0.05; **P < 0.01

In the present study, mice were immunized with MOG_35‐55_ peptide to induce EAE model. As shown in Table 1 and Figure 1, the incidence in Fasudil‐MNCs group (80%) was decreased as compared with that in PBS‐MNCs group (100%). In PBS‐MNCs group, mean onset date was 10.4 ± 1.4, mean maximum symptom score was 2.4 ± 0.9. The bilateral intranasal instillation of Fasudil‐modified MNCs delayed onset (12.5 ± 0.9, *P* < 0.01), and declined maximum clinical score (1.0 ± 0.8, *P* < 0.01). Simultaneously, the weight loss in Fasudil‐MNCs group was smaller than that of PBS‐MNCs group.

**Table 1:** The clinical evaluation of EAE mice

Group	n	Incidence (%)	Mean onset date	Mean score of maximal symptom
PBS‐MNCs	10	100	10.4 ± 1.4	2.4 ± 0.9
Fasudil‐MNCs	10	80	12.5 ± 0.9^a^	1.0 ± 0.8^a^

EAE, experimental autoimmune encephalomyelitis; MNCs, mononuclear cells.

Chronic EAE was induced in C57BL/6 mice with MOG_35‐55_. Fasudil‐treated MNCs were administrated by bilateral intranasal instillation with 3 × 10^6^ cells/unilateral nasal cavity on day 3 postimmunization (p.i.) and day 11 p.i. PBS‐treated MNCs were set up as control in a similar manner. Data are presented as mean ± SEM. Differences are analyzed using Student's *t* test.

a *P* < 0.01.

### 3.2 Fasudil‐modified MNCs inhibits inflammation and improves demyelination in spinal cords

**Figure 2:**
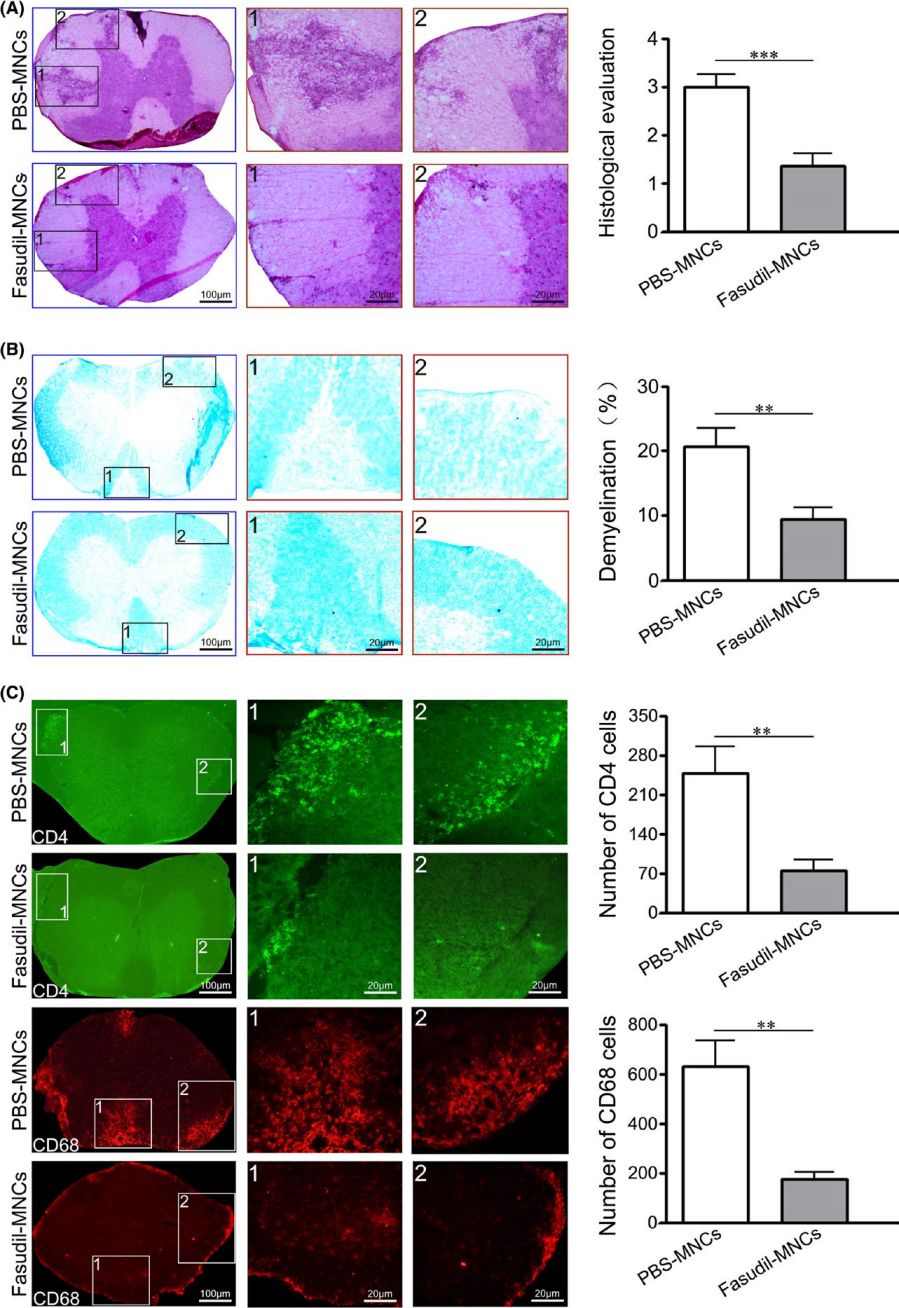
Fasudil‐modified mononuclear cells (MNCs) inhibit inflammation and improve myelination in spinal cords. Chronic experimental autoimmune encephalomyelitis was induced in C57BL/6 mice with MOG_35‐55_. Fasudil‐modified MNCs were administrated by bilateral intranasal instillation with 3 × 10^6^ cells/unilateral nasal cavity on day 3 postimmunization (p.i.) and 11 p.i. Phosphate buffered solution (PBS)‐treated MNCs were set up as control in a similar manner. On day 28 p.i., spinal cords were used for H&E, luxol fast blue and immunohistochemistry staining. A, Inflammation stained with H&E stain. B, Demyelination stained with luxol fast blue. Total white matter in luxol fast blue was manually outlined, and pixel area (%) of demyelination in total white matter was calculated by Image‐Pro Plus software. C, Infiltration of CD4^+^ T cells and CD68^+^ macrophage. Results are shown as mean ± SEM of five mice in each group. Differences are analyzed using Student's *t*‐test. **P < 0.01; ***P < 0.001

In the spinal cord of EAE, the inflammatory infiltration CNS and demyelination are major pathological manifestation. As shown in Figure 2A,B, nasal delivery of Fasudil‐modified MNCs obviously reduced inflammatory infiltration (*P* < 0.001) and improved demyelination (*P* < 0.01) in spinal cords compared with PBS‐treated MNCs group.

Results from immunofluorescence staining indicated that spinal cord of mice in PBS‐MNCs group contained higher invasion of CD4^+^ T cells and CD68^+^ macrophages, which obviously was declined by nasal delivery of Fasudil‐modified MNCs compared with PBS‐MNCs group (Figure 2C, all *P* < 0.01).

### 3.3 Fasudil‐modified MNCs regulate peripheral T cells and macrophages

**Figure 3:**
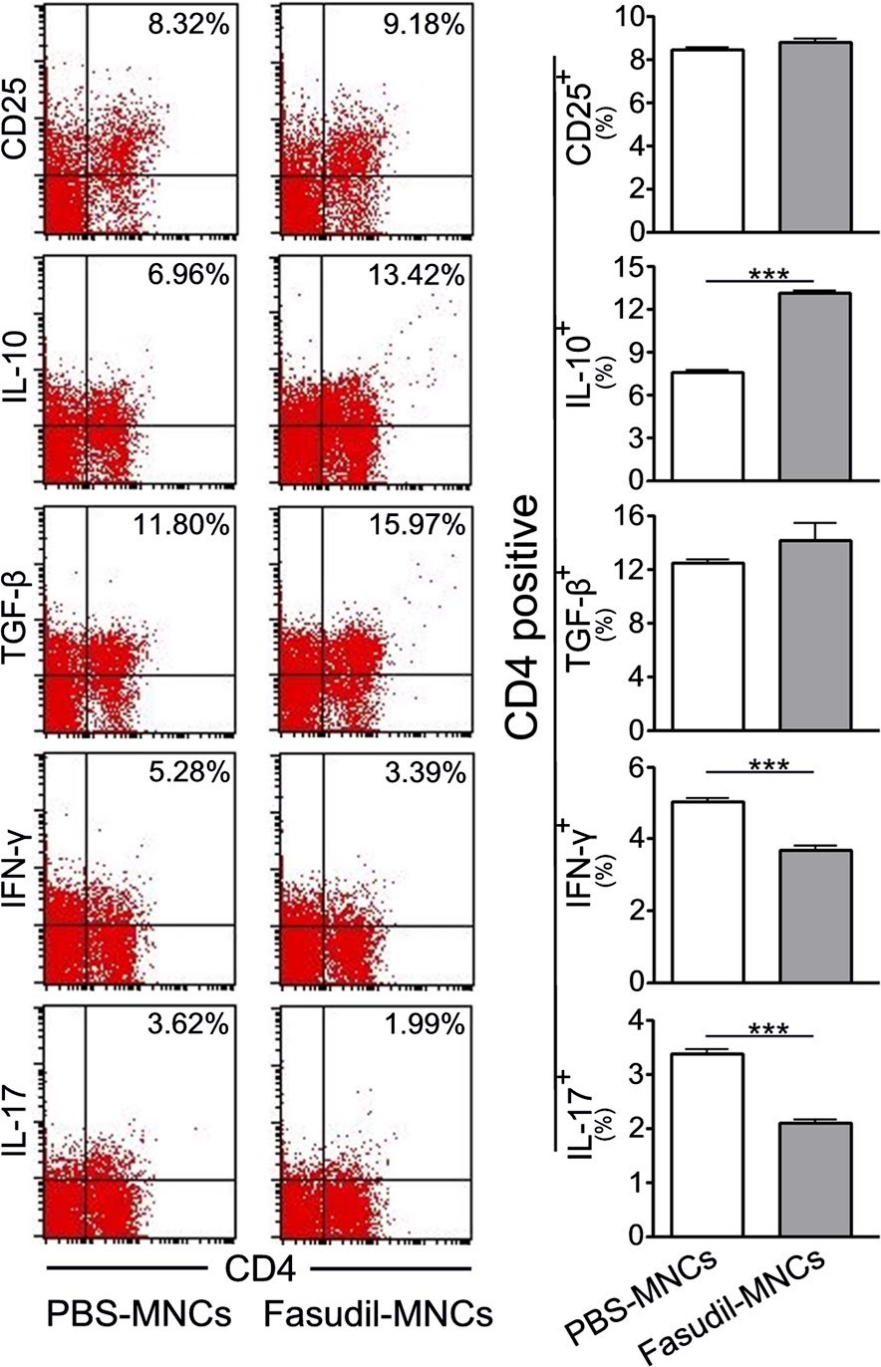
Fasudil‐treated mononuclear cells (MNCs) affect T‐cell differentiation. Chronic experimental autoimmune encephalomyelitis (EAE) was induced in C57BL/6 mice with MOG_35‐55_. Fasudil‐modified MNCs were administrated by bilateral intranasal instillation with 3 × 10^6^ cells/unilateral nasal cavity on day 3 postimmunization (p.i.) and 11 p.i. Phosphate buffered solution (PBS)‐treated MNCs were set up as control in a similar manner. On day 28 p.i., splenic MNCs were prepared and stained with anti‐CD4, CD25, IL‐10, TGF‐β, IL‐17, IFN‐γ, and subsets of T cells were analyzed using flow cytometry. Quantitative results are analyzed for five mice in each group. Results are expressed as the percentage of double positive cells in four‐quadrant diagram. Differences are analyzed using Student's *t*‐test. ***P < 0.001

The activation of the peripheral immune system is the initiating factor in the pathogenesis of EAE, which can infiltrate to CNS and lead to immune attacks and meylin damage. To detect the effect of Fasudil‐modified MNCs on immunomodulatory in EAE mice, the subsets of T cells and macrophages were analysed by flow cytometry. Percentages of CD4^+^ T cells expressing IL‐10 were elevated, while percentages of CD4^+^ T cells expressing IL‐17 and IFN‐γ were decreased in Fasudil‐MNCs group, as compared with PBS‐MNCs group (Figure 3, *P* < 0.001 respectively). There was no difference on subsets of CD4^+^CD25^+^ and CD4^+^TGF‐β^+^ T cells between PBS‐MNCs group and Fasudil‐MNCs group (Figure 3).

**Figure 4:**
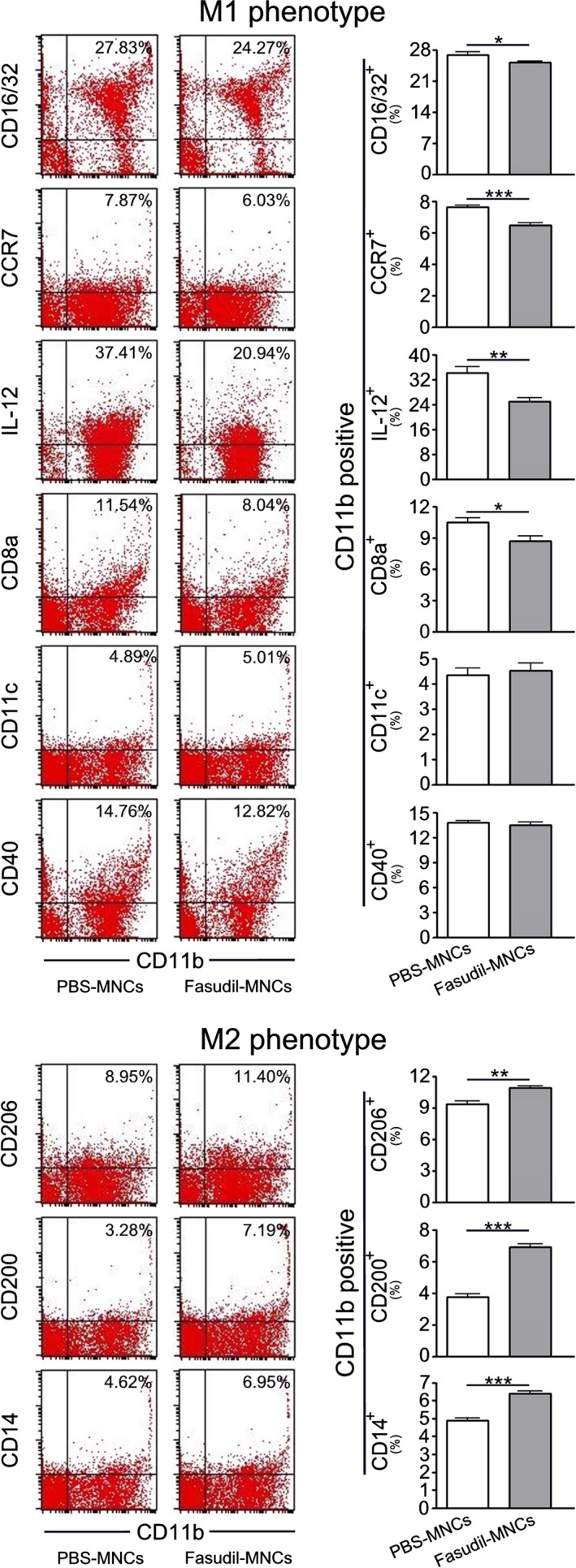
Fasudil‐modified mononuclear cells (MNCs) affect macrophages differentiation. Chronic experimental autoimmune encephalomyelitis (EAE) was induced in C57BL/6 mice with MOG_35‐55_. Fasudil‐modified MNCs were administrated by bilateral intranasal instillation with 3 × 10^6^ cells/unilateral nasal cavity on day 3 postimmunization (p.i.) and 11 p.i. Phosphate buffered solution (PBS)‐treated MNCs were set up as control in a similar manner. On day 28 p.i., splenic MNCs were prepared and stained with anti‐CD11b, CD16/32, CCR7, IL‐12, CD8a, CD11c, CD40, CD206, CD200, CD14, and subsets of macrophages were analyzed using flow cytometry. Quantitative results are analyzed for five mice in each group. Results are expressed as the percentage of double positive cells in four‐quadrant diagram. Differences are analyzed using Student's *t*‐test. *P < 0.05; **P < 0.01; ***P < 0.001

We next observed the changes of macrophage populations in spleen by flow cytometry. As shown in Figure 4, intranasal instillation of Fasudil‐modified MNCs inhibited the subsets of CD11b^+^ macrophages expressing M1 markers CD16/32 (*P* < 0.05), CCR7 (*P* < 0.001), IL‐12 (*P* < 0.01) and CD8a (*P* < 0.05) and increased the subsets of CD11b^+^ macrophages expressing M2 markers CD206 (*P* < 0.01), CD200 (*P* < 0.001) and CD14 (*P* < 0.001), as compared with PBS‐treated MNCs control.

**Figure 5:**
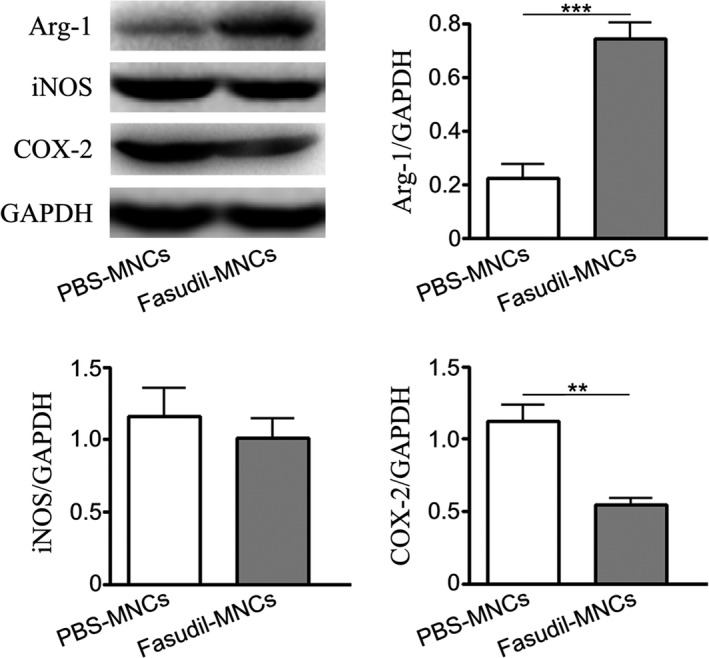
Fasudil‐modified mononuclear cells (MNCs) may shift M1 to M2 phenotype in spinal cord. Chronic experimental autoimmune encephalomyelitis (EAE) was induced in C57BL/6 mice with MOG_35‐55_. Fasudil‐modified MNCs were administrated by bilateral intranasal instillation with 3 × 10^6^ cells/unilateral nasal cavity on day 3 postimmunization (p.i.) and 11 p.i. Phosphate buffered solution (PBS)‐treated MNCs were set up as control in a similar manner. On day 28 p.i., spinal cords were collected and western blot was carried out to compare expression of iNOS, COX‐2 and Arg‐1 protein. Results are shown as mean ± SEM of five mice in each group. Differences are analyzed using Student's *t*‐test. **P < 0.01; ***P < 0.001

Based on literature review, iNOS, COX‐2 and Arg‐1 are three representatives of M1 and M2 macrophages. We further measured the expression of iNOS, COX‐2 and Arg‐1 in spinal cords by western blot. As shown in Figure 5, nasal delivery of Fasudil‐modified MNCs significantly suppressed expression of COX‐2 (*P* < 0.01) and increased expression of Arg‐1 (*P* < 0.001). There was no significant difference on the expression of iNOS between Fasudil‐MNCs group and PBS‐MNCs group in spinal cord.

### 3.4 Fasudil‐modified MNCs inhibit inflammatory responses in EAE

**Figure 6:**
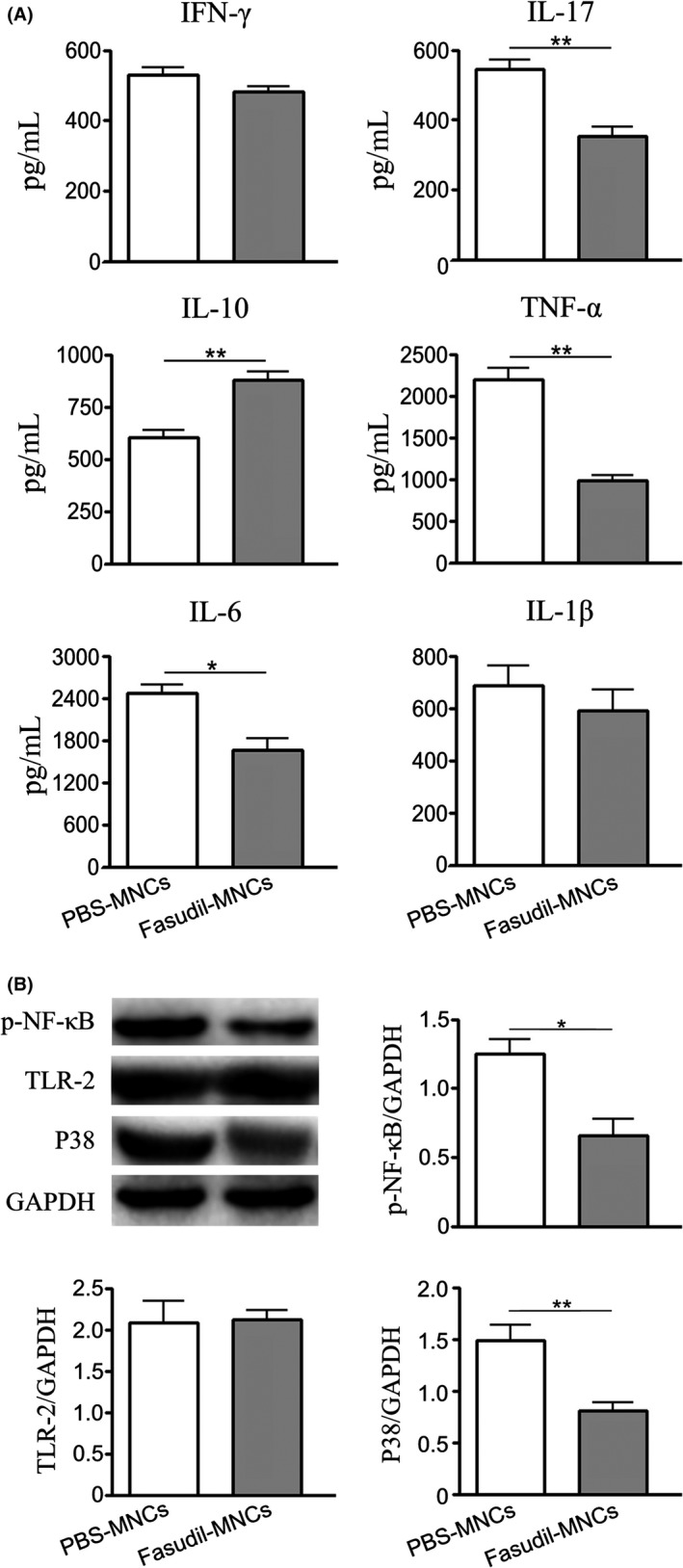
Fasudil‐modified mononuclear cells (MNCs) inhibit inflammatory responses in experimental autoimmune encephalomyelitis (EAE) mice. Chronic EAE was induced in C57BL/6 mice with MOG_35‐55_. Fasudil‐modified MNCs were administrated by bilateral intranasal instillation with 3 × 10^6^ cells/unilateral nasal cavity on day 3 postimmunization (p.i.) and 11 p.i. Phosphate buffered solution (PBS)‐treated MNCs were set up as control in a similar manner. On day 28 p.i., splenic MNCs and spinal cords were prepared and the supernatants were collected. A, The levels of IFN‐γ, IL‐17, IL‐10, TNF‐α, IL‐6, IL‐1β were measured by enzyme linked immunosorbent assay (ELISA) kits. B, The expression of TLR2, p‐NF‐κB and P38 by western blot. Results are shown as mean ± SEM of five mice in each group. Differences are analyzed using Student's *t*‐test. *P < 0.05; **P < 0.01

Neuroinflammation played an important role in the pathogenesis of EAE, involving the release of inflammatory cytokines and the activation of signaling pathways. To understand whether Fasudil‐modified MNCs influence inflammatory reaction, we measured the levels of inflammatory and antiinflammatory cytokines, such as IFN‐γ, IL‐17, IL‐10, TNF‐α, IL‐6, IL‐1β by ELISA kits. As expected, the levels of IL‐17, TNF‐α and IL‐6 were suppressed (Figure 6A, *P* < 0.05 and *P* < 0.01, respectively), while the level of IL‐10 was enhanced in Fasudil‐MNCs group (Figure 6A, *P* < 0.05) compared with PBS‐MNCs group. Given that TLR2‐p‐NF‐κB‐P38 signaling pathway contributes to inflammatory responses, we measured the changes of TLR2‐p‐NF‐κB‐P38 axis by western blot. Nasal delivery of Fasudil‐modified MNCs effectively inhibited the expression of p‐NF‐kB and P38 in spinal cord (Figure 6B, *P* < 0.05 and *P* < 0.01, respectively). However, there was no significant difference on the expression of TLR‐2 protein in spinal cords between two groups (Figure 6B).

### 3.5 Fasudil‐modified MNCs induce the expression of neurotrophic factors in spinal cord

**Figure 7:**
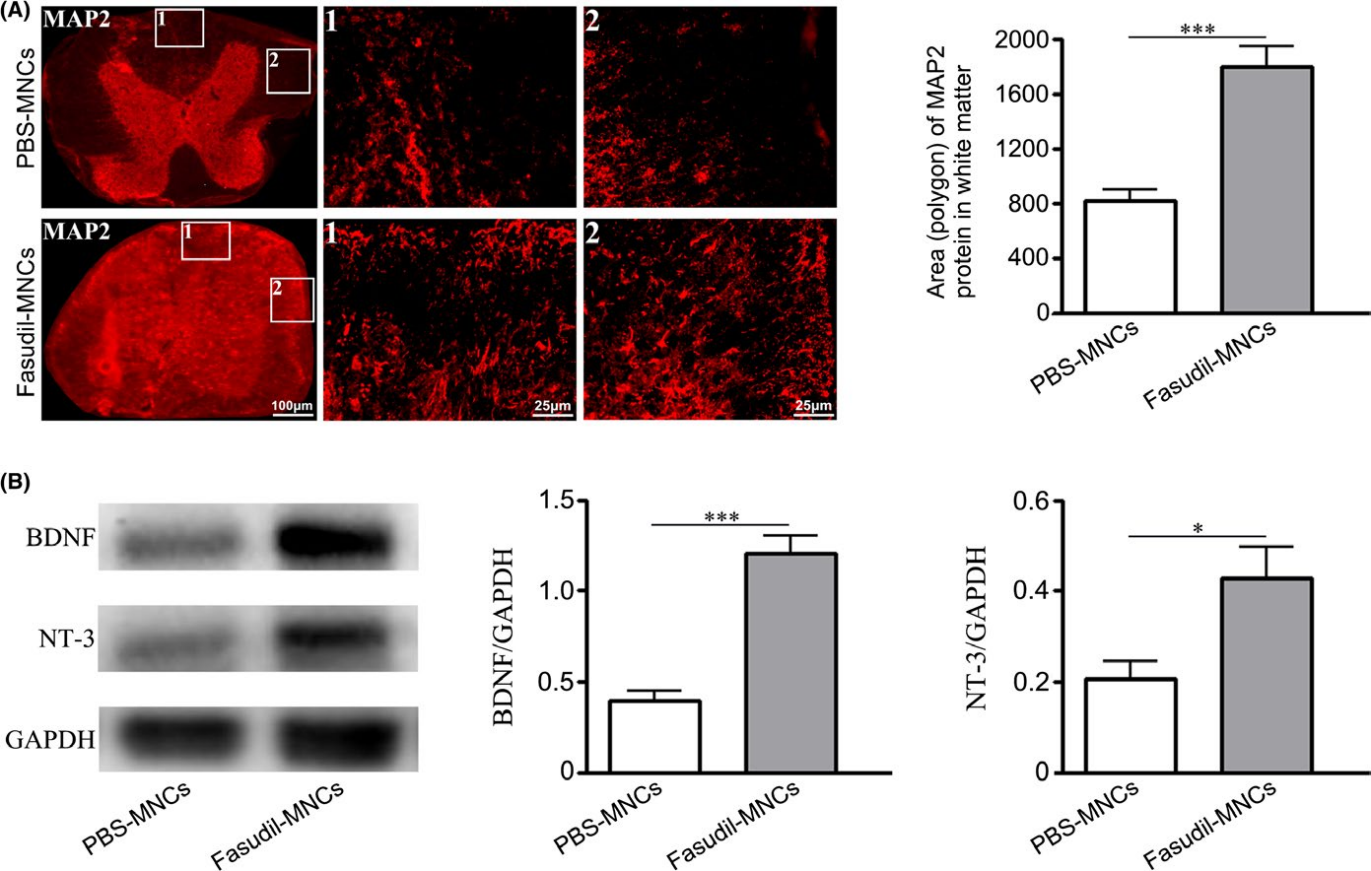
Fasudil‐modified mononuclear cells (MNCs) has a neuroprotective effect in experimental autoimmune encephalomyelitis (EAE) mice. Chronic EAE was induced in C57BL/6 mice with MOG_35‐55_. Fasudil‐treated MNCs were administrated by bilateral intranasal instillation with 3 × 10^6^ cells/unilateral nasal cavity on day 3 postimmunization (p.i.) and 11 p.i. Phosphate buffered solution (PBS)‐treated MNCs were set up as control in a similar manner. On day 28 p.i., spinal cords were prepared. A, The expression of MAP2 performed by immunohistochemistry staining. B, The expression of brain derived neurotrophic factor (BDNF) and NT‐3 were determined by western blot. Results are shown as mean ± SEM of five mice in each group. Differences are analyzed using Student's *t*‐test. *P < 0.05; ***P < 0.001

It is speculated that the improvement of inflammatory microenvironment can reduce neuron damage. As shown in Figure 7, MAP‐2^+^ neurons in spinal cord were enhanced in mice treated with Fasudil‐modified MNCs (*P* < 0.001), accompanied by the increase of BDNF and NT‐3 (*P* < 0.001 and *P* < 0.05, respectively), as compared with control mice. These results indicate that Fasudil‐modified MNCs exhibited neuroprotective effect possibly by inhibiting inflammatory microenvironment and inducing neurotrophic factors.

## 4 DISCUSSION

Our previous studies found that Fasudil, when injected intraperitoneally in early and late stages of EAE induction, ameliorated the clinical severity of EAE, improved demyelination and inhibited the infiltration of inflammatory cells.^17^ However, the clinical limitation of Fasudil in treating MS is also obvious, because of the vasodilation effect, smaller safety window for long‐term use, poor oral bioavailability and no oral drug.^19^, ^23^ Thus, we used nasal delivery of MNCs modified by Fasudil in vitro for cell therapy in EAE. The results show that nasal delivery of Fasudil‐modified MNCs delayed onset of EAE, ameliorated severity of symptoms and improved demyelination, providing a novel strategy for cell therapy in the treatment of EAE/MS.

Intranasal administration has aroused wide interest in the past few years. A series of studies have reported the efficacy and safety of intranasal route in the treatment of nervous system diseases.^25^ Cells can enter straight through the olfactory nerve and olfactory tract into the cranial cavity and connect with mitral cell and plexus cells in the olfactory bulb by synapsis, which form the olfactory mucosa epithelium pathway.^26^, ^27^ This route minimizes the distribution and first pass effect of immune cells in peripheral organs and allows rapid drug delivery directly from nasal mucosa to the brain. There are currently more than 300 clinical trials under way in the USA alone using intranasal administration.^28^, ^29^, ^30^ Our previous study showed that the treatment effect of nasal administration was superior to that of intraperitoneal injection in PD model mice and nasal delivery of the Fasudil derivative inhibited the development of EAE.^22^, ^29^ In the present research, nasal delivery of Fasudil‐modified MNCs exhibited therapeutic potential by regulating the peripheral immune responses and inhibiting the central inflammatory microenvironment, which provide a safe and effective option for the treatment of MS.

The activation of macrophage/microglia is considered to be an important factor in causing demyelination and neurodegeneration.^17^, ^18^ Axonal damage may be positively correlated with the activation of inflammatory macrophage/microglia.^31^, ^32^ Macrophage is usually classified into Ml and M2 phenotype based on different functional and phenotypes. The M1 phenotype of macrophage/microglia generally expresses oxidative stress products and inflammatory factors and M2 phenotype of macrophage/microglia inhibits inflammatory responses and promotes tissue repair.^10^, ^33^

According to a large amount of literature in the relevant field of M1 and M2 macrophage, we selected the markers of macrophage M1 polarization, that is, iNOS, IL‐12 and CD16/32, as well as the markers of M2 polarization, that is, Arg‐1, CD206 and CD200.^33^ Using flow cytometry, we found that CD16/32, IL‐12, CCR7, CD8a on CD11b^+^ macrophages were downregulated and CD206, CD200, CD14 on CD11b^+^ macrophages were enhanced in Fasudil‐MNCs group. The experimental results showed that Fasudil‐modified MNCs partially shifted inflammatory M1 to antiinflammatory M2 macrophage in splenocytes. It is generally believed that microglia are specialized macrophage and the major immune effector cell in CNS, which also have obvious heterogeneity.^34^ Microglia plays a multitude of detrimental and beneficial actions on the aggression of myelin sheath and the recovery tissue damage in MS.^33^, ^35^ Therefore, we focus on microglia, expecting to enhance the positive effect and inhibit its negative effect, which will undoubtedly play a great role in disease treatment. Several studies reports that post mortem brain and spinal tissue from MS patients contain increased levels of COX‐2 in association with microglia, macrophages and oligodendrocytes.^36^ COX‐2 leads to proinflammatory eicosanoids, and stimulates cytokine production and activation of microglia and astrocytes, thereby contributing to MS pathology.^37^, ^38^ Arg‐1, a cytosolic enzyme, is important in the repair of damaged tissue by generating putrescine that has pro‐repair properties. The expression of Arg‐1 is strongly induced in microglia/macrophage by antiinflammatory cytokines IL‐4 and IL‐13, and therefore is often used to define M2 polarization state.^39^ Arg‐1 also competes with iNOS for l‐arginine, reducing the production of nitric oxide, a free radical with diverse cytotoxic and harmful inflammatory actions.^40^ In our current study, Fasudil‐modified MNCs suppressed COX‐2 expression and increase Arg‐1 expression in spinal cords. Therefore, nasal delivery of Fasudil‐modified MNCs has therapeutic potential in EAE possibly through mediating the polarization of macrophage/microglia in both spleen and CNS.

Multiple sclerosis exhibits myelin and nerve damage caused by cascade reaction of autoreactive T cells. Th1 and Th17 cells can activate macrophage/microglia and inflammatory responses by secreting IFN‐γ and TNF‐α, shape MS plaque and result in myelinoclasis and axonal degeneration.^41^ Th2 cells can induce M2 macrophages to suppress inflammation, which improves the severity of EAE.^42^ As expected, our results also displayed that Fasudil‐modified MNCs decreased the population of CD4^+^ T cells expressing IL‐17 and IFN‐γ, and triggered a stronger CD4^+^ T cells expressing IL‐10 in spleen of EAE mice, revealing that Fasudil‐modified MNCs could regulate T cells to achieve antiinflammatory and immunomodulatory effects in the peripheral immune system.

Inflammation constitutes the common channel of neurodegeneration and is an essential pathogenesis in many nervous system diseases, such as MS, trauma and stroke. 43, 44, 45The following question is whether Fasudil‐modified MNCs can influence the inflammatory microenvironment within the CNS. NF‐κB, a protein of transcriptional activation, plays an important role in many pathological processes such as inflammation, immune response, cell proliferation and differentiation.^46^, ^47^ NF‐κB can promote the expression of adhesion molecules and mediate the infiltration of peripheral inflammatory cells into CNS.^48^ Our result showed that the expression of NF‐κB was inhibited in spinal cord of mice treated with Fasudil‐modified MNCs. P38, a transfer of extracellular stimulation signals to the nucleus, is one of the important signal transduction systems that induce cellular biological responses, promoting the release of cytokines such as TNF‐α, IL‐1β, NO.^49^, ^50^ In spinal cords, Fasudil‐modified MNCs suppressed the expression of P38. Furthermore, Fasudil‐modified MNCs decreased the production of inflammatory cytokines IL‐17, TNF‐α, IL‐6 and increased antiinflammatory IL‐10 production in culture supernatant of splenocytes.^51^ Our data suggested that inhibiting the activation of NF‐κB‐p38 signaling pathway was a possible target of Fasudil‐modified MNCs in the treatment of EAE. In addition, NF‐κB is a key transcription factor that is related to the activation of M1 macrophage, while P38 could mediate inflammatory injury under microglia activation.^52^, ^53^ Moreover, inflammation and oxidative stress also enhanced the expressions of NF‐κB and P38, which eventually formed a vicious circle.^54^

Besides immunomodulation and antiinflammation, Fasudil‐modifed MNCs have capacity to induce the expressions of neurotrophic factor BDNF and NT‐3 in spinal cord, which can promote the remyelination. BDNF is well‐known to promote myelin repair in the brain,^55^, ^56^ axonal growth^57^ and neuronal survival and differentiation.^58^ BDNF is also shown to stimulate oligodendrocyte differentiation and maturation.^59^ NT‐3 is a growth factor that modulates oligodendrocyte differentiation and maturation^60^ and augments remyelinating and immunomodulatory capacity of neural stem cells.^61^ In fact, Fasudil‐modified MNCs raises the level of MAP‐2^+^ neurons in spinal cord, which should be related to the upregulation of BDNF and NT‐3 expression.

## 5 CONCLUSION

In conclusion, our findings provide evidence that nasal delivery of Fasudil‐modified MNCs delays the onset and ameliorates the severity in EAE mice, accompanied by the improvement of demyelination. Fasudil‐modified MNCs regulates immune responses, improves the inflammatory microenvironment and induces neurotrophic factors possibly through the re‐balance of Th17 T cells and Th2 T cells, the polarization of M1 to M2 phenotype, and the up regulation of BDNF/NT‐3. Nnasal delivery of Fasudil‐modified MNCs represents a safe and effective cell therapeutic strategy to MS and/or other related disorders.

## CONFLICT OF INTEREST

The authors declare no conflict of interest.
